# Inhibitory control deficits in patients with mesial temporal lobe epilepsy: an event-related potential analysis based on Go/NoGo task

**DOI:** 10.3389/fneur.2023.1326841

**Published:** 2024-01-08

**Authors:** Chenjing Shao, Desheng Li, Xu Zhang, Feng Xiang, Xi Zhang, Xiangqing Wang

**Affiliations:** ^1^Medical School of Chinese PLA, Beijing, China; ^2^Department of Neurology, The Second Medical Centre, National Clinical Research Centre for Geriatric Diseases, Chinese PLA General Hospital, Beijing, China; ^3^Department of Neurology, The First Medical Centre of Chinese PLA General Hospital, Beijing, China

**Keywords:** temporal lobe epilepsy, impulsivity, inhibitory control, event-related potential, Go/NoGo task

## Abstract

**Objective:**

Neuropsychiatric comorbidities are common among patients with mesial temporal lobe epilepsy (MTLE). One of these comorbidities, impulsivity, can significantly impact the quality of life and prognosis. However, there have been few studies of impulsivity in these patients, and the existing findings are inconsistent. The present study investigates impulsivity in MTLE patients from the perspective of inhibitory control and its underlying processes using event-related potentials (ERPs) initiated using a Go/NoGo task.

**Methods:**

A total of 25 MTLE patients and 25 age-, gender-, and education-matched healthy controls (HCs) completed an unequal visual Go/NoGo task. Different waveforms as well as behavioral measures were analyzed between Go and NoGo conditions (N2d and P3d). Impulsivity was also assessed using self -rating scales, and clinical variables that may be related to ERPs were explored.

**Results:**

Compared with HCs, MTLE patients exhibited significantly longer reaction time (RT) (*p* = 0.002) and lower P3d especially at the frontal electrode sites (*p* = 0.001). In the MTLE group, the seizure frequency (*p* = 0.045) and seizure types (*p* < 0.001) were correlated with the P3d amplitude. A self-rated impulsivity assessment revealed that MTLE patients had higher non-planning (*p* = 0.017) and total scores (*p* = 0.019) on the BIS-11 as well as higher DI (*p* = 0.010) and lower FI (*p* = 0.007) on the DII.

**Conclusion:**

The findings demonstrate that the presence of inhibitory control deficits in patients with MTLE are characterized by deficits in the late stage of inhibition control, namely the motor inhibition stage. This study improves our understanding of impulsivity in MTLE patients and suggests that ERPs may constitute a sensitive means of detecting this trait.

## 1 Introduction

Apart from the epileptic focus, there are also impairments in other brain regions and networks in epilepsy (1). Therefore, epilepsy, and particularly mesial temporal lobe epilepsy (MTLE), is often accompanied by various neuropsychological disorders (2). Previous studies have shown that neuropsychological comorbidities are prevalent in over half of MTLE patients (3). Among these comorbidities, impulsivity has gained increasing attention (4–6). Increased impulsivity has been reported in both generalized epilepsy and focal epilepsy (7). The positive correlation between seizure frequency and impulsivity has suggested that seizures and impulsivity may share common neural network damage. Moreover, the hyperexcitability of epileptic focal neurons can also lead to increased impulsivity, rendering epilepsy patients susceptible to high impulsivity (8).

Impulsivity is not a singular concept, rather it encompasses a broad range of actions that are poorly conceived, prematurely expressed, unduly risky, or inappropriate to the situation and it often leads to undesirable outcomes (9, 10). In patients with epilepsy, heightened impulsivity not only contributes to poor social adaptability, but also renders individuals more susceptible to various seizure-inducing factors, such as substance abuse, sleep deprivation, and nonadherence (7, 8, 11, 12). Additionally, studies have reported a positive correlation between high impulsivity and suicidality in patients with epilepsy (4, 5, 13). Considering the potential impact of impulsivity on quality of life and prognosis, more research on impulsivity, particularly in those with MTLE, is necessary. Thus, MTLE remains an important area of research as well as a challenge in the clinical practice.

Studies on impulsivity of epilepsy patients have primarily focused on juvenile myoclonic epilepsy, yielding a virtually consistent conclusion of increased impulsivity (6, 11, 14–18). Findings of studies on MTLE patients have been inconsistent. For instance, Sang-Ahm et al. (7) reported that MTLE patients exhibited lower attentional, motor, and total scores and higher non-planning scores on the Barratt Impulsiveness Scale (BIS-11) compared with HCs. Conversely, Rzezak et al. (18) found no difference in any of the three BIS-11 subscales between MTLE patients and HCs, while in another study using behavioral tasks researchers reported inhibition control impairments (19). Additionally, both Labudda et al. (16) and Ruken et al. (20) reported decision-making deficits on the Iowa Gambling Task in MTLE patients.

The reasons behind these differences merit study. They may involve the multidimensional nature of impulsivity and the limitations of each assessment instrument (21). The BIS-11 might not capture the specific impulsivity dimensions increased in MTLE patients, although it effectively detects high impulsivity in JME. Self-rating scales may be susceptible to subjective factors since they depend on self-perception. However, MTLE patients commonly experience cognitive impairments (22), which could lead to reduced self-awareness.

Given the aforementioned reasons, there is a need for more empirical research on impulsivity in MTLE patients. Inhibitory control deficits are one manifestation of high impulsivity that refer to the inability to suppress prepotent responses and can be measured by behavioral tasks (23). In individuals with MTLE, various clinical manifestations of inhibitory control deficits have been reported, such as poor medical compliance (24), deficits in attentional control (25), expressions of “venting” and “behavioral disengagement” (26), obsessive-compulsive symptoms (27), and interictal episodes of aggression (28).

The Go/NoGo task, a classic paradigm for assessing inhibition control, requires participants to respond quickly and accurately to one type of stimulus (Go) while inhibiting their response to a different type of stimulus (NoGo) (29, 30). This task mainly elicits two event-related potentials (ERPs): an early negative deflection around 200–400 ms (N2) post-stimulus onset and a late positive component peaking around 300–600 ms (P3) (31). Both components provide insights into the time course of response inhibition and execution with high temporal resolution (30, 32). While there are existing studies on P3 in MTLE patients (33–35), they predominantly employ oddball tasks, where P3 primarily reflects cognitive processes such as attention and working memory, rather than serving as an assessment of inhibitory control (36, 37). Go/NoGo tasks combined with ERP measurements have been used to study inhibitory control in various pathologies, but they have only rarely been used in MTLE patients.

The main purpose of this study was to investigate inhibitory control characteristics in MTLE patients by using the Go/NoGo task and its evoked ERP components (N2 and P3). Additionally, two widely used impulsivity self-rating scales, the BIS-11 and the Dickman’s Impulsivity Inventory (DII), were administered to participants. Considering previous evidence of frontal lobe dysfunction (19, 38) and some aspects of high impulsivity on behavioral tasks in MTLE patients (16, 19, 20), we expected that there would be intergroup differences in the ERP measures.

## 2 Methods

### 2.1 Participants

Participants were recruited from the Epilepsy Clinic of the Department of Neurology of the First Medical Center of the Chinese PLA General Hospital between January 2022 and December 2022.

The inclusion criteria for MTLE patients were as follows: (i) age between 16 and 55 years; (ii) diagnosed with MTLE according to the new classifications of seizures and epilepsy of the International League Against Epilepsy (39); (iii) completion of a long-term scalp video-EEG and a 3 T structural MRI scan; (iv) right-handed; (v) inclusive of all genders.

Patients were excluded if they met any of the following criteria: (i) major psychiatric or neurological disorders, except for epilepsy; (ii) severe cognitive impairment (MMSE < 20); (iii) substance abuse or dependence, other than antiseizure medications (ASMs), within the past 6 months; (iv) severe visual impairment; (v) experienced focal to bilateral tonic-clonic seizures (FBTCS) within the past 72 h.

HCs were recruited through an advertisement. All participants were right-handed, had normal or corrected-to-normal vision, and did not have a prior history of neurological or psychiatric illness.

Written informed consent was obtained from each participant or their guardian. The study protocol was approved by the Ethics Committee of Chinese PLA General Hospital (S2023-129-01).

### 2.2 Clinical information collection

A qualified neurologist conducted a detailed medical history and neurological examination of each participant, including age of seizure onset, disease duration, seizure types, seizure frequency, current antiseizure medication (ASM) regimen, and results from EEG and MRI.

Cognition, depression, and anxiety were assessed using the Montreal Cognitive Assessment (MoCA), Self-Rating Depression Scale (SDS), and Self-Rating Anxiety Scale (SAS), respectively.

### 2.3 Self-assessment of impulsivity

The Barratt Impulsiveness Scale (BIS-11) (40) consists of 30 items and measures impulsivity across three dimensions: attentional impulsivity (eight items), motor impulsivity (11 items), and non-planning impulsivity (11 items). Each item is scored on a range of 1–4, with the total score ranging from 30 to 120. Scores for each subscale and the total score were analyzed, with higher scores indicating greater impulsivity.

The DII assesses impulsivity across two distinct domains: dysfunctional impulsivity (DI), which is associated with harmful risk-taking behavior, and functional impulsivity (FI), which relates to positive characteristics (41). It comprises a total of 23 items (11 for FI and 12 for DI), with respondents answering either yes or no. Scores of both FI and DI were analyzed.

### 2.4 Go/NoGo task

A classic visual Go/NoGo task with frequent-Go/rare-NoGo stimuli was presented in yellow against a black background at the center of a computer screen placed 100 cm away from the participant. The “Go” stimulus was any of the Arabic numerals from “1” to “9,” while the “NoGo” stimulus was the number “0.”

**Figure 1:**
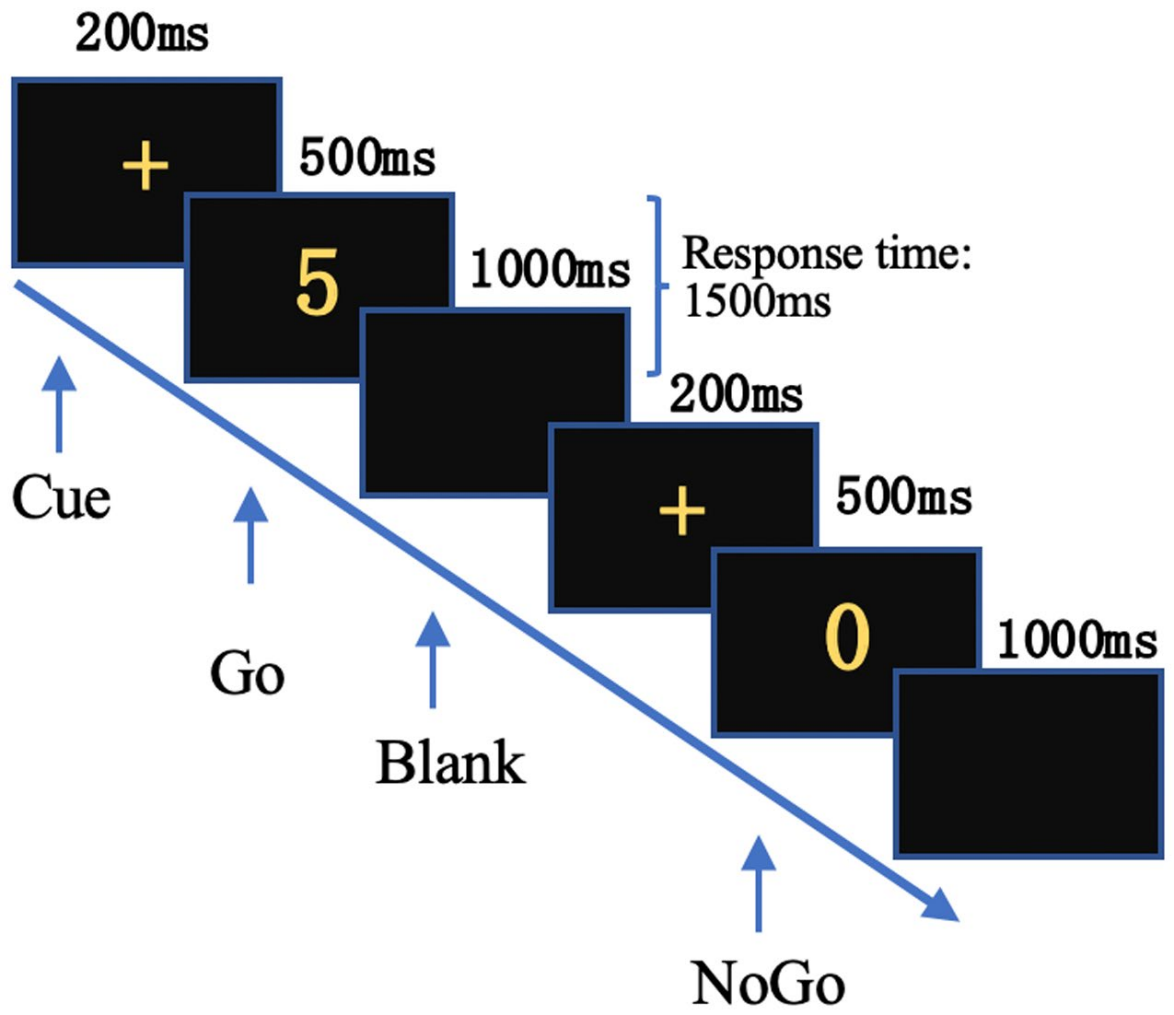
Time course of the Go/NoGo task. A cue “+” was presented first, followed by the Go or NoGo stimulus. Subjects were allowed to respond until the next “+” appeared.

Stimuli were presented by E-prime 2.0. The experiment began with 30 trials for practice, followed by 300 trials in the experimental session. The initial 30 stimuli consisted exclusively of “Go” stimuli to increase participants’ responsiveness, however, these were excluded from subsequent analyses. The remaining 270 trials included randomly distributed “Go” and “NoGo” stimuli (189 “Go” stimuli and 81 “NoGo” stimuli) with a fixed stimulus interval of 1700 ms (Figure 1).

Participants were instructed to press the space bar rapidly and accurately with their right index finger when a “Go” stimulus appeared and to refrain from responding when a “NoGo” stimulus appeared. The Go accuracy (Go-ACC), NoGo accuracy (NoGo-ACC), and reaction time (RT) to Go stimuli were analyzed.

### 2.5 EEG recording and analysis

EEG recording was conducted using a NeuroScan SynAmps amplifier (Brain Products Inc.) with 64 Ag/AgCl electrodes placed in a cap (Neuroscan Quikcap) according to the 10–20 International System. The electrodes were referenced to the whole-brain average. An electrode located between FPz and Fz served as the ground. Vertical eye movements were recorded from above and below the right eye, and horizontal eye movements were recorded from the outer canthi of both eyes. Impedance was maintained below 5 kΩ. The sampling rate was set to 1,000 Hz, with an online bandpass filter of 0.1–100 Hz.

The EEGLAB2021 toolbox was used for off-line data processing. A bandpass filter with a range of 0.1–30 Hz was applied. The electrodes were re-referenced to the average of mastoids (42). Eye movements were identified by independent component analysis; subsequently, they were removed manually. The EEG data were segmented into epochs of 1,000 ms (200 ms pre- and 800 ms post-stimulus) with a baseline of 200 ms prior to stimuli. Epochs with amplitude exceeding ±70 μV were automatically rejected from further processing. Additionally, epochs containing epileptiform discharges were also removed. After artifact rejection, in the MTLE group, the number of analyzed epochs for Go trials was 130.60 (SD = 20.49), and for NoGo trials, it was 60 (SD = 9.90). For HCs, the corresponding values were 145.72 (SD = 19.95) for Go trials and 63.80 (SD = 9.10) for NoGo trials. There were no significant differences in epoch numbers observed between the two groups for both Go and NoGo trials.

Time windows for N2 and P3 were determined by visually inspecting of grand average waveforms across the midline. The time window for N2 was set at 200–370 ms, while for P3 it was set at 300–600 ms. Peaks of each component in individual ERP waveforms were first automatically detected by EEGLAB and then manually checked and adjusted.

**Figure 2:**
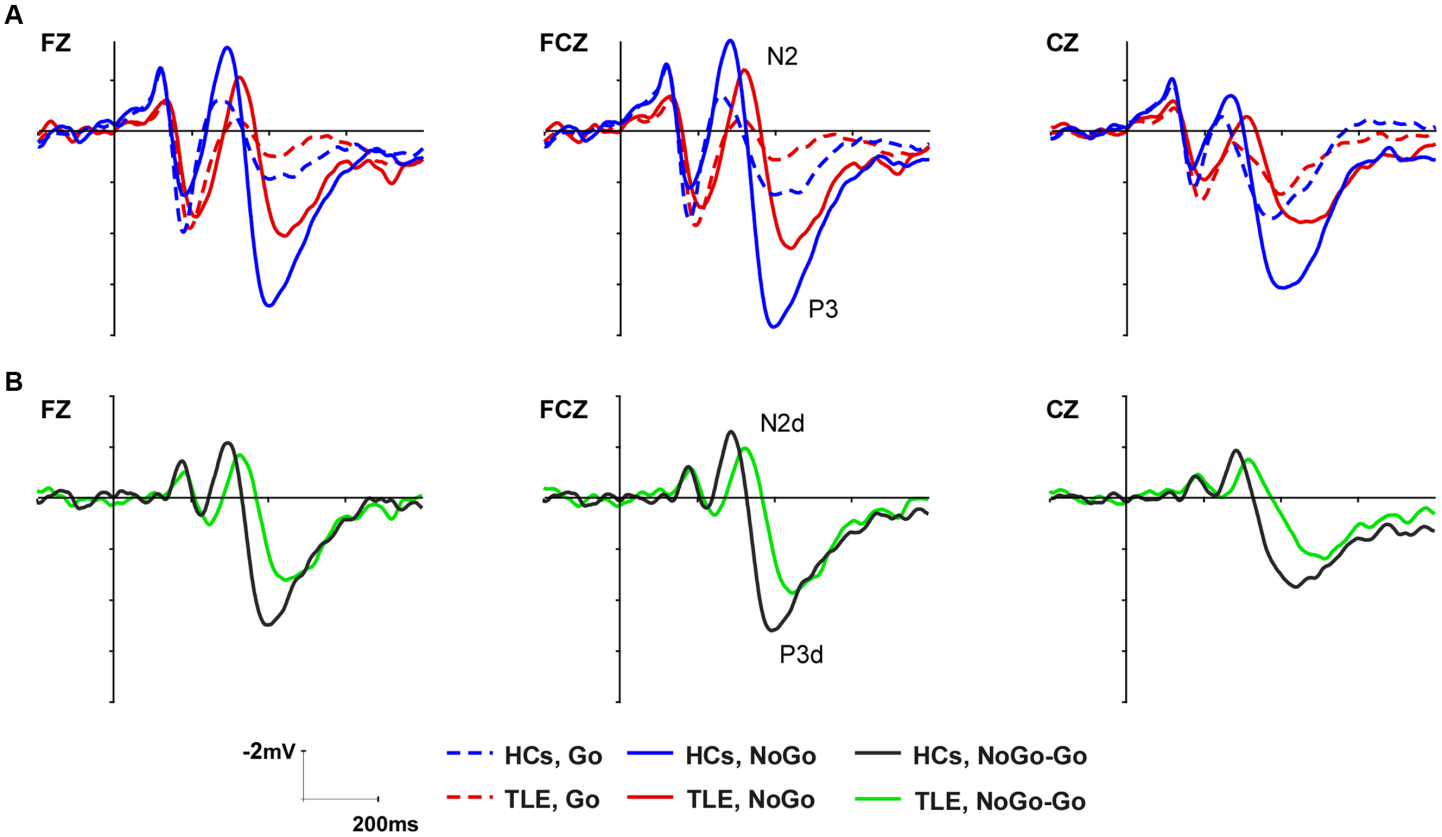
Grand average ERP waveforms of the N2 and P3 components at Fz, FCz, and Cz in the two groups **(A)**. Difference waveforms computed by subtracting ERPs to Go trials from ERPs to NoGo trials **(B)**.

According to the grand-averaged ERP waveforms, the latency and amplitude of the MTLE group were smaller and later in both Go and NoGo conditions than in HCs (Figure 2A). In order to highlight the NoGo effect – characterized by a more prominent N2 and P3 during NoGo trials compared to Go trials and considered indicative of the neural response associated with inhibitory processes – distinct waveforms as N2d and P3d were computed by subtracting ERPs to Go trials from ERPs to NoGo trials (43, 44). The peak amplitude and latency of N2, P3, N2d, and P3d were analyzed at Fz, FCz, and Cz as these electrodes showed the highest modulation of these components (45) (Figure 2B).

### 2.6 Statistical analysis

Statistical analysis was conducted using SPSS 23.0 (IBM Corp). Normality and homogeneity of the data were assessed using the Shapiro–Wilk test and Levene’s test, respectively. Normally distributed data were presented as mean and standard deviation (SD), while non-normally distributed data were presented as median and interquartile range (IQR). Categorical data were reported as numbers and percentages. Intergroup comparisons of the demographic variables and scale scores were conducted using Student’s *t*-test, Mann–Whitney U test, and chi-square test, as appropriate. RT was compared using ANOVA. Regrading accuracy rates (ACCs), generalized linear equations (GLE) were employed due to their departure from normality, with the condition (Go vs. NoGo) as a within-subject factor and the group (TLE vs. HCs) as a between-subject factor.

N2 and P3 amplitude and latency were analyzed using three-way repeated measures ANOVA (RM-ANOVA) with within-subject factors of condition (Go vs. NoGo) and site (Fz, FCz, and Cz), and the between-subject factor of group (MTLE vs. HCs). For N2d and P3d amplitude and latency, a two-way RM-ANOVA was conducted, with the site (Fz, FCz, and Cz) as within-subject factors, and the group (MTLE vs. HCs) as a between-subject factor. The degrees of freedom were adjusted using the Greenhouse–Geisser correction for violations of sphericity. *Post hoc* pairwise comparisons with the Bonferroni correction (corrected *p*-values reported) were utilized to further examine significant main effects and interaction effects. Partial eta-squared (*η*_p_^2^) was used as a measure of effect size.

Considering that SDS, SAS, and MoCA scores may contribute to changes in ERPs (3, 46), stepwise linear regressions were conducted with the ERPs showing significant group differences as dependent variables and with the SDS score, SAS score, MoCA score, and group as predictors.

In the MTLE group, Spearman’s correlation analyses were performed between the P3b amplitude and clinical characteristics. Notably, in terms of seizure type, FBTCS designates individuals who not only experience relatively frequent focal onset, such as the commonly reported aura of epigastric rising sensation followed by complex partial seizures, but also occasionally have secondary bilateral tonic-clonic seizures (47). N2d was not included due to the lack of significant between-group differences.

The significance level was set at *p* < 0.05 for all analyses.

## 3 Results

### 3.1 Cognitive and psychological assessments

A total of 95 MTLE patients and 52 HCs completed the questionnaires (Table 1). MTLE patients exhibited significantly lower scores on the MoCA (*t* = −3.811; *p* < 0.001) and higher scores on the SDS (*t* = 2.436; *p* = 0.019) and SAS (*t* = 3.134; *p* = 0.003) than the HCs (Table 2).

**Table 1:** Demographic and neuropsychological data of the MTLE group and HCs who completed the impulsivity questionnaires.

	MTLE (*n* = 95)	HCs (*n* = 52)	*p* value
Men, *n* (%)	52 (54.7%)	30 (57.7%)	*p* = 0.862
Age (years), mean (SD)	30.59 (9.57)	29.35 (10.05)	*p* = 0.461
Education (years), median (IQR)	12.00 (6.00)	15.00 (7.00)	*p* = 0.176
**BIS-11**			
Attentional, mean (SD)	16.85 (3.23)	16.10 (3.34)	*p* = 0.182
Motor, mean (SD)	21.37 (4.17)	20.83 (3.92)	*p* = 0.433
Non-planning, mean (SD)	27.24 (4.74)	25.19 (5.25)	*p* = 0.017
Total, mean (SD)	66.33 (10.15)	62.12 (10.30)	*p* = 0.019
**DII**			
FI, mean (SD)	4.93 (2.77)	6.17 (2.46)	*p* = 0.007
DI, mean (SD)	4.35 (3.00)	3.02 (2.86)	*p* = 0.010

BIS-11, the Barratt impulsiveness scale-11; DII, the Dickman’s impulsivity inventory; DI, dysfunctional impulsivity; FI, functional impulsivity.

**Table 2:** Demographic and neuropsychological data of the MTLE group and HCs who participated in the Go/NoGo task.

	MTLE (*n* = 25)	HCs (*n* = 25)	*p* value
Men, *n* (%)	15 (60%)	14 (56%)	0.774
Age (years), mean (SD)	31.80 (9.62)	29.88 (10.45)	0.502
Education (years), median (IQR)	12.00 (6.50)	15.00 (5.00)	0.073
MoCA	24.68 (3.52)	28.00 (2.57)	0.001
SDS, mean (SD)	47.60 (10.05)	40.45 (10.69)	0.019
SAS, mean (SD)	44.85 (11.16)	36.20 (8.11)	0.003
**Behavior performance**			
Go-ACC, median (IQR)	1.00 (0.01)	1.00 (0.01)	0.251
NoGo-ACC, median (IQR)	0.90 (0.18)	0.90 (0.13)	0.864
Go-RT (s), mean (SD)	417.08 (46.52)	375.24 (41.79)	0.002

MTLE, mesial temporal lobe epilepsy; HCs, healthy controls; MoCA, Montreal cognitive assessment; SDS, self-rating depression scale; SAS, self-rating anxiety scale; ACC, accuracy; RT, reaction time.

### 3.2 Impulsivity self-assessment

Compared with HCs, MTLE patients showed higher non-planning (*t* = 2.413, *p* = 0.017) and total scores (*t* = 2.382, *p* = 0.019) on the BIS-11 as well as higher DI (*t* = 2.611, *p* = 0.010) and lower FI (*t* = −2.712, *p* = 0.007) on the DII (Table 1).

### 3.3 Behavioral performance analysis

Based on the principle of voluntary participation, 55 subjects also participated in the Go/NoGo task and EEG signal acquisition. Among them, three patients and two HCs were excluded due to too many EEG artifacts. Finally, behavioral and electrophysiological measures were analyzed in 25 MTLE patients and 25 HCs. Clinical variables related to epilepsy in the MTLE group are summarized in Table 3.

**Table 3:** Epilepsy-related characteristics of the MTLE group (*n* = 25).

	*n* or mean	Proportion (%) or median (range)
Age at onset of epilepsy (years)	22.24 (12.46)	23 (2–47)
Disease duration (years)	9.64 (10.76)	5 (0.5–45.5)
**Seizure types**		
Focal onset only	10	40%
FBTCS	15	60%
Seizure frequency > 3 Times/month	17	68%
Presence of epileptiform discharge	16	64%
**MTS**		
Without MTS	6	24%
Right MTS	4	16%
Left MTS	9	36%
Bilateral MTS	6	24%
**ASM regime**		
Untreated	3	12%
Monotherapy	6	24%
Two ASMs	11	44%
Three or more ASMs	5	20%

FBTCS, focal to bilateral tonic-clonic seizure; MTS, mesial temporal sclerosis; ASM, antiseizure medication.

The MTLE group exhibited significantly longer RT for correct Go trials than HCs (*F* = 11.192; *p* = 0.002). Regarding the ACC, GLE revealed a significant main condition effect (OR = 1.154; *p* < 0.001), indicating higher accuracy for Go trials than for NoGo trials in both groups. The main effect of group was not significant (OR = 1.007; *p* = 0.864) (Table 2).

### 3.4 ERP data

#### 3.4.1 N2 peak amplitude

Significant main effects emerged for condition [*F* (1,48) = 31.585, *p* < 0.001, *η*_p_^2^ = 0.397] and site [*F* (2,96) = 18.197, *p* < 0.001, *η*_p_^2^ = 0.275], accompanied by a notable interaction of condition*site [*F* (2,96) = 7.436, *p* = 0.004, *η*_p_^2^ = 0.134]. Post-hoc comparisons indicated that both Go-N2 and NoGo-N2 exhibited the least negativity at Cz (Go: MTLE: −0.11 μV, HCs: −1.19 μV; NoGo: MTLE: −1.49 μV, HCs: −2.42 μV). NoGo-N2 consistently showed greater negativity than Go-N2 across all sites (*p* < 0.001). Despite a trend towards less negative N2 in the MTLE group, the main group effect did not reach statistical significance [*F* (1,48) = 3.528, *p* = 0.066, *η*_p_^2^ = 0.068].

#### 3.4.2 N2 peak latency

The significant main effect of group [*F* (1,48) = 33.181, *p* < 0.001, *η*_p_^2^ = 0.409] indicated an earlier N2 in the HCs for both conditions. Additionally, there were significant main effects of condition [*F* (1,48) = 20.808, *p* < 0.001, *η*_p_^2^ = 0.302], site [*F* (2,96) = 40.394, *p* < 0.001, *η*_p_^2^ = 0.457], and a significant interaction of condition*site [*F* (2,96) = 4.825, *p* = 0.021, *η*_p_^2^ = 0.091]. Post-hoc analyses revealed a significantly later NoGo-N2 across all sites compared to Go-N2 (*p* < 0.05). The earliest N2 for both conditions occurred at Cz (Go: MTLE: 274.44 ms, HCs: 239.84 ms; NoGo: MTLE: 304.92 ms, HCs: 266.12 ms).

#### 3.4.3 P3 peak amplitude

Significant main effects were found for group [*F* (1,48) = 9.796, *p* = 0.003, *η*_p_^2^ = 0.169] and condition [*F* (1,48) = 110.854, *p* < 0.001, *η*_p_^2^ = 0.698], along with interactions of condition*group [*F* (1,48) = 10.123, *p* = 0.003, *η*_p_^2^ = 0.174] and condition*site [*F* (2,96) = 25.439, *p* < 0.001, *η*_p_^2^ = 0.346]. Post-hoc comparisons revealed higher NoGo-P3 than Go-P3 in both groups at all sites (*p* < 0.001). Go-P3 peaked at Cz (MTLE: 3.23 μV, HCs: 4.15 μV), while NoGo-P3 was maximal at FCz (MTLE: 5.19 μV, HCs: 8.08 μV). The MTLE group exhibited significantly lower NoGo-P3 (*p* < 0.001), with no significant between-group difference for Go-P3.

#### 3.4.4 P3 peak latency

A significant main effect of group [*F* (1,48) = 7.696, *p* = 0.008, *η*_p_^2^ = 0.138] indicated a delayed P3 in the MTLE group for both conditions. The main effect of site [*F* (2,96) = 9.090, *p* < 0.001, *η*_p_^2^ = 0.159] and the interaction of condition*site [*F* (2,96) = 13.555, *p* < 0.001, *η*_p_^2^ = 0.220] were also significant. Post-hoc analysis indicated that the earliest Go-P3 occurred at Cz (MTLE: 412.56 ms, HCs: 389.04 ms), with no significant differences observed in NoGo-P3 across sites. In both groups, NoGo-P3 was delayed only at Cz, compared to Go-P3 (*p* < 0.001).

#### 3.4.5 Difference waves

**Figure 3:**
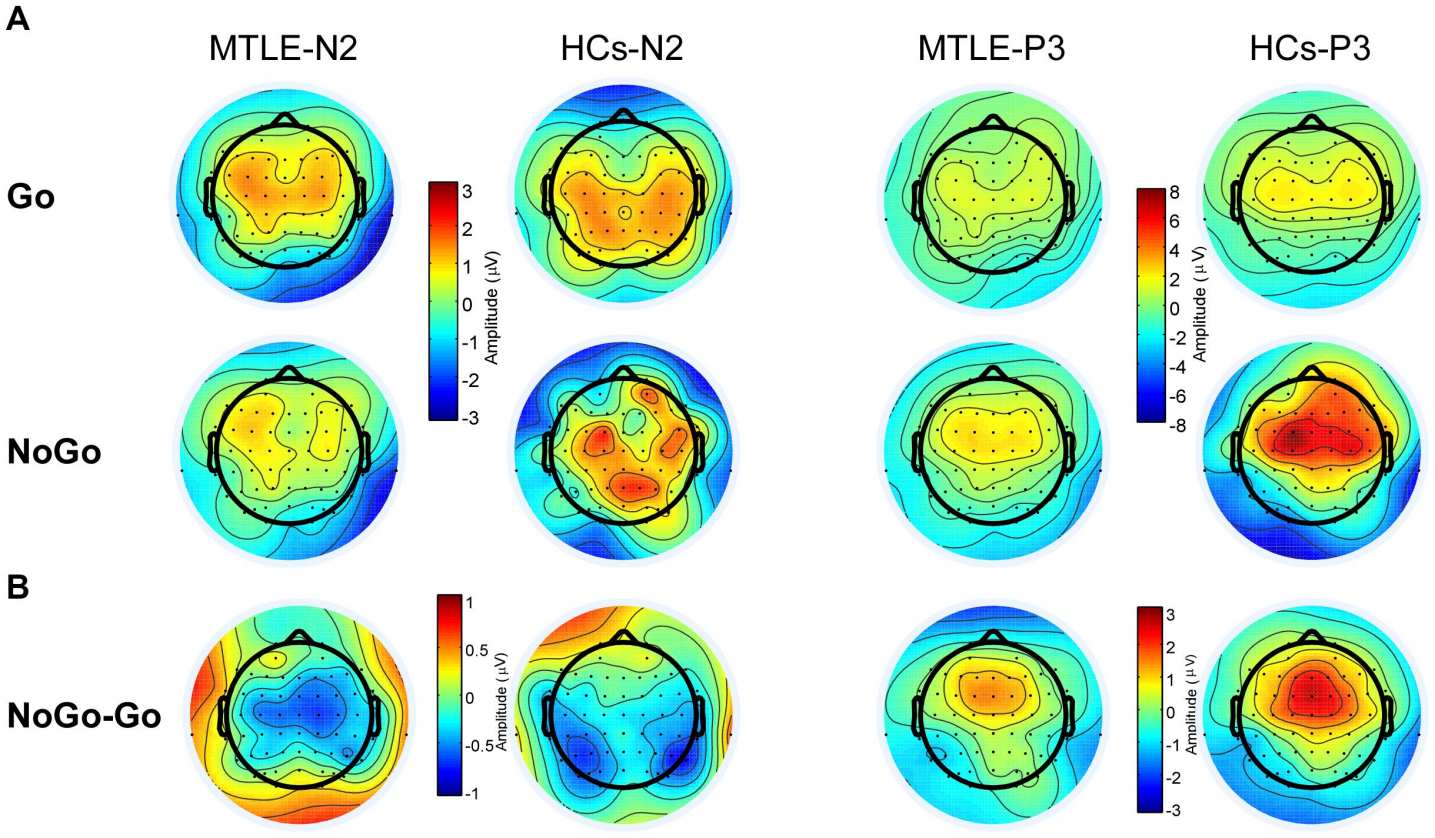
Topographic Maps of N2 (200–370 ms) and P3 (200–370 ms) Components **(A)** and N2d (200–370 ms) and P3d (200–370 ms) in the two groups **(B)**.

As shown in the grand-average ERP waveform (Figure 2A) and topographic maps (Figure 3A), the N2 and P3 components in the MTLE group exhibited smaller and delayed responses under both conditions compared to the HCs. And these inter-group differences in N2 and P3 latencies under both conditions, as well as in NoGo-P3 amplitudes, were statistically significant. To intuitively evaluate inhibitory control, our focus centered on the NoGo effect, measured by N2d and P3d (Figures 2B, 3B).

For the P3d amplitude, the main effect of group [*F* (1,48) = 10.123, *p* = 0.003, *η*_p_^2^ = 0.174] was significant. *Post hoc* analysis showed that the MTLE group had lower P3ds at Fz (*p* = 0.001), FCz (*p* = 0.008), and Cz (*p* = 0.019) than those of HCs. The main effect of site [*F* (2,96) = 25.439 *p* < 0.001, *η*_p_^2^ = 0.346] was also significant. Post-hoc analysis showed that P3d was biggest at FCz in both groups.

For P3d latencies, the main effect of site was significant [*F* (2,96) = 13.555, *p* < 0.001, *η*_p_^2^ = 0.220]. In both groups, the NoGo latencies at Fz and FCz were shorter than those of Go.

For the N2d amplitude, the main effect of site was significant [*F* (2,96) = 7.436, *p* = 0.004, *η*_p_^2^ = 0.134]. In both groups, the most negative N2d was observed at FCz (MTLE: −2.26 μV, control: −2.16 μV). No other significant main effect or interaction of group*site was found (Table 4).

**Table 4:** The amplitude and latency of N2d and P3d of the MTLE group and HCs.

	N2d		P3d	
	MTLE (*n* = 25)	HCs (*n* = 25)	MTLE (*n* = 25)	HCs (*n* = 25)
**Amplitude (μV)**				
Fz	−1.98 (2.27)	−1.97 (2.80)	2.75 (2.35)	5.19 (2.63)
FCz	−2.26 (2.10)	−2.16 (3.15)	3.12 (2.82)	5.36 (2.94)
Cz	−1.38 (1.84)	−1.23 (2.68)	1.45 (2.11)	3.17 (2.85)
**Latency (ms)**				
Fz	16.12 (38.18)	9.76 (28.43)	−8.08 (72.39)	−12.62 (80.53)
FCz	17.04 (40.47)	9.16 (33.04)	−8.88 (77.31)	−3.96 (74.03)
Cz	30.48 (42.29)	26.28 (34.94)	33.88 (55.58)	32.19 (79.09)

MTLE, mesial temporal lobe epilepsy; HCs, healthy controls.

Considering the potential impact of SDS, SAS, and MoCA on measures of inhibitory control, we next investigated the contribution of the group and the aforementioned scores on P3d changes using linear regression. The P3d amplitude at Fz (where inter-group differences were most significant) was a dependent variable, whereas the group and three scale scores were independent variables. The results showed that the group (*β* = 1.976, *p* = 0.025) could significantly predict the P3d amplitude, whereas the MoCA (*β* = 0.028, *p* = 0.819), SDS (*β* = 0.039, *p* = 0.492), and SAS (*β* = −0.068, *p* = 0.258) scores did not. This suggests that even when controlling for emotional cognition and other factors, patients with MTLE still exhibited lower P3d than HCs.

### 3.5 Correlations

Spearman correlation revealed two significant correlations between the P3d (Fz) amplitude and clinical variables in the MTLE group. Specifically, the P3d amplitude was negatively correlated with seizure types (*R* = −0.623, *p* = 0.001) and seizure frequency (*R* = −0.404, *p* = 0.045).

## 4 Discussion

The objective of this study was to investigate whether patients with MTLE exhibit impaired inhibition control as assessed by behavioral and ERP measures in a Go/NoGo task. Compared with HCs, the MTLE group demonstrated longer RT to Go stimuli. In terms of ERPs, the MTLE group displayed smaller P3d. These findings provide evidence of abnormal response inhibition processing in patients with MTLE.

### 4.1 Self-assessment of impulsivity

Partially in line with the findings of Sang-Ahm et al. (7), we observed a higher non-planning score but also a higher total score on BIS-11 in MTLE patients. This differs from studies conducted in JME patients, where specific domains of impulsivity measured by BIS-11 showed an increase (6, 11, 18). Future research may be needed to explore the practicality of this scale in such patients. Currently, there are few studies using the DII to measure impulsivity in patients with epilepsy. In the present study, we observed increased functional impulsivity and decreased dysfunctional impulsivity, which suggests that the impulsivity impairment in MTLE patients is selective.

### 4.2 Behavioral performance and inhibition control

The Go/NoGo task challenges one’s ability to withhold a response, particularly when Go stimuli appear more frequently than NoGo stimuli (30). In the present study, Go-ACC was higher than NoGo-ACC in both groups, indicating that the imbalanced Go/NoGo task successfully established a difficult-to-inhibit prepotent response (48). Furthermore, analyzing the pattern of performance differences can provide insights into potential deficits (49). For instance, a high proportion of NoGo errors, with or without shortened RT, is indicative of inhibition control deficits. A high proportion of Go errors may suggest lapses of attention. However, when accompanied by prolonged RT, they may also indicate the presence of inhibitory control deficits (49). In the present study, an intergroup comparison only revealed prolonged RT in the MTLE group. One possible explanation for this discrepancy is that the task used in our study was simple enough to enable patients to compensate for perceived or actual inhibitory control deficits by slowing down the processing speed, resulting in similar ACC between the groups (50).

### 4.3 Electrophysiology and inhibition control

The Go/NoGo task involves various cognitive components, including working memory, stimulus-driven attention, conflict detection, error monitoring, top-down control, and response inhibition (51). N2 and P3 represent different cognitive processes, however, consensus has not been reached regarding their specific functions (52). Go-N2 is generally considered to reflect stimulus evaluation and categorization (53, 54). Barry and De Blasio (54) proposed that together with Go-P3, it may also be involved in response preparation and execution. Based on this, Go-P3 may represent the outcome of response selection processes following an earlier phase of N2 (54–56). Several studies support the idea that NoGo-N2 is associated with conflict detection between response and non-response and the decision to inhibit the response (30, 32, 53, 57, 58). Nogo-P3 is traditionally linked to motor inhibition processes (31, 58). Several studies suggest that it may also be involved in post-response stages, such as evaluation or monitoring of inhibition, error detection, and preparation for upcoming trials (59, 60). To highlight the “NoGo effect” and more precisely illustrate the frontal inhibitory processing function, most models use difference waves, specially N2d and P3d. N2d is commonly utilized as a neuromarker for conflict detection and P3d as a neuromarker of motor inhibition. Consistent with the patterns typically observed in the Go/NoGo task, we detected larger N2 and P3 under the NoGo condition than under the Go condition in the frontal and central regions (known as the NoGo effect) (48, 56, 61). These findings could be attributed to the activation of brain regions associated with inhibitory control (62). Specifically, the primary generators of NoGo-N2 have consistently been located in the anterior cingulate cortex (23, 63), while NoGo-P3 has been shown to originate from the left lateral orbitofrontal cortex, a region with reciprocal projections to the anterior cingulate cortex (62). The presence of the NoGo effect suggests that MTLE patients are able to activate and execute inhibitory processes.

The smaller P3d amplitude specifically observed at Fz in MTLE patients relative to HCs suggests that they may have frontal lobe dysfunction, which can hinder their ability to allocate adequate neural resources to the motor inhibition subprocess, consequently resulting in a diminished P3b. Reduced P3b has been observed in various disorders characterized by inhibitory control impairments, such as alcohol-dependency, obesity, schizophrenia, ADHD, exercise addiction, and others (64–67).

In general, inhibitory control operates hierarchically, with the prefrontal cortex (PFC) playing a pivotal role in regulating subordinate neural structures. In detail, cortical regions most often involved in response inhibition tasks encompass the right inferior frontal cortex (IFC), OFC, anterior cingulate cortex, ventrolateral PFC, dorsolateral PFC, parietal cortex, Supplementary Motor Area (SMA), pre-SMA, pre-motor cortex, and insula. Subcortically, structures consist of the Subthalamic Nucleus and striatum, participating in the hyperdirect pathway and fronto-striatal loop, respectively (68). Several investigations into impaired performance in Go/NoGo task have identified the medial PFC as a primary contributor (69–71). Anomalies in the metabolism and function of the anterior cingulate cortex (72, 73), as well as structural and functional alterations in the OFC (74, 75), have been reported in MTLE patients. Electro-clinical findings also suggest the potential involvement of the OFC in seizure activity (76). Indeed, neuroimaging studies have revealed that MTLE is a network disorder, characterized by profound alterations in both localized and distributed networks (38). These alternations may also involve other structures within the inhibitory control network. Regarding the hippocampus, no studies have been found indicating its direct involvement in response inhibition. Benoit et al. (77) reported that, during the process of memory inhibition, the hippocampus is regulated by the dorsolateral PFC.

Moreover, abnormal neural metabolite levels in MTLE may impact inhibitory function. The neurotransmitters closely associated with epileptic seizures, glutamate and γ-aminobutyric acid (GABA) (78, 79), have been shown to be linked to impulsive behavior and inhibitory control in both healthy individuals and those with neuropsychiatric conditions (80, 81). Altered levels of these substances have also been reported in MTLE patients. For instance, He et al. (82) reported a reduced GABA/Cr ratio in the anterior cingulate cortex of individuals with MTLE compared to HCs, using quantitative magnetic resonance spectroscopy (MRS). Additionally, Malthankar et al. (83) found glutamate dehydrogenase enzyme deficiency in the anterolateral temporal neocortex and hippocampal tissues of MTLE patients, which may lead to potential glutamate accumulation. Although, as we know, no studies have reported abnormal glutamate concentrations in the frontal lobe of MTLE patients, it remains plausible that its involvement occurs through intricate brain networks or seizure propagation (84).

Above all, alterations in the structure, function, and metabolism, particularly those involving the frontal lobe, may contribute to the inhibitory control deficits in those patients.

An additional noteworthy finding in this study is that, even with the inclusion of the MoCA, SDS, and SAS scores in the regression model, significant intergroup differences were observed, suggesting that the inhibitory control deficit in MTLE patients may be modulated by other factors. Therefore, clinical factors related to impulsivity were further explored.

### 4.4 Clinical moderators of impulsivity

Seizure frequency is the most reported clinical variable potentially associated with impulsivity. Positive correlations between seizure frequency and impulsivity have been reported in idiopathic generalized epilepsy (6, 8) and unclassified adult epilepsy (13). In the current study, we found that higher seizure frequency was associated with lower P3d. These findings support the hypothesis proposed by Barratt et al. (85) that seizures and impulsivity may share the same mechanism of neuronal hyperexcitability, leading to high impulsivity in patients with epilepsy.

We also found a negative correlation between the P3b amplitude and seizure types as patients with FBTCS were more likely to have smaller P3d. This suggests that these patients may have more severe and extensive brain network alterations and dysfunction, highlighting the need for increased attention to this subgroup.

Although we did not find any correlation between ASMs and behavioral/ERP measures, the impact of ASMs on impulsivity cannot be ignored. Most ASMs, including valproate/divalproex sodium, lamotrigine, oxcarbazepine, carbamazepine, topiramate, and phenytoin, have been shown to be effective in alleviating impulsive aggressive behavior, except for LEV (13). Regarding the therapy regimen, although Sang-Ahm et al. (13) found a positive correlation between ASM polytherapy and impulsivity, they attributed it to patients having more severe drug-resistant epilepsy rather than adverse reactions to polytherapy.

### 4.5 Limitations

The current study contributes to our understanding of inhibitory control in MTLE patients; nevertheless, it is important to acknowledge several limitations. First, the study exclusively centers on MTLE, lacking a disease control group, like individuals with frontal lobe epilepsy. Second, due to limitations in sample size, we did not perform a more detailed classification of MTLE, such as distinctions based on various etiologies, left versus right hippocampal sclerosis, and refractory versus non-refractory states. Third, the relationship between inhibitory control and suicide risk was not investigated (86). Lastly, the inclusion of a wide variety of ASMs in the patient sample, without disqualification based on type and dosage, limits the ability to draw conclusions about the effects of specific ASMs on inhibitory control. The correlation analysis included only 25 cases of MTLE patients, which could affect the statistical power.

## 5 Conclusion

The present study shows the presence of inhibitory control deficits in patients with MTLE characterized by lower P3d, specifically at the Fz electrode. This suggests that during the late stage of inhibition control, namely the motor inhibition stage, MTLE patients are unable to mobilize sufficient neurons, which may be due to frontal lobe dysfunction. Moreover, the inhibitory control deficits of MTLE patients are also related to clinical factors, such as seizure frequency and type. In addition, impulsivity self-assessment scale data indicated that MTLE patients also have a certain degree of impairment in other aspects of impulsivity. This study is a preliminary exploration. In the future, research with larger sample sizes will be necessary to validate our findings and further evaluate the relationships among clinical features, ASMs, and impulsivity. More research in electrophysiology, such as time-frequency analysis, source localization, and studies on brain networks, as well as fMRI would also further show the processing mechanisms of inhibitory control in patients with MTLE.

## Data availability statement

The raw data supporting the conclusions of this article will be made available by the authors, without undue reservation.

## Ethics statement

The studies involving humans were approved by The Ethics Committee of Chinese PLA General Hospital. The studies were conducted in accordance with the local legislation and institutional requirements. Written informed consent for participation in this study was provided by the participants’ legal guardians/next of kin.

## Author contributions

CS: Investigation, Methodology, Visualization, Writing – original draft, Writing – review & editing, Formal analysis, Software. DL: Writing – review & editing, Supervision. XuZ: Writing – review & editing, Data curation. FX: Writing – review & editing, Investigation. XiZ: Funding acquisition, Resources, Writing – review & editing. XW: Writing – review & editing, Project administration.
